# Toward person-centred measures of contraceptive demand: a systematic review of the relationship between intentions to use and actual use of contraception

**DOI:** 10.12688/gatesopenres.15078.2

**Published:** 2024-06-25

**Authors:** Victoria Boydell, Kelsey Quinn Wright, Shatha Elnakib, Christine Galavotti

**Affiliations:** 1 University College London, London, England, UK; 2 University of Helsinki, Helsinki, Uusimaa, Finland; 3 Johns Hopkins University, Baltimore, Maryland, USA; 4 Bill & Melinda Gates Foundation, Seattle, Washington, USA

**Keywords:** Systematic review, contraception, intention, preferences

## Abstract

**Background:**

Understanding people’s interest in using modern contraception is critical to ensuring programs align with people’s preferences and needs. Current measures of demand for contraception are misinterpreted. More direct measures of intention to use (ITU) contraception do exist but remain underexplored. This systematic review examines the relationship between intention to use and actual use of contraception.

**Methods:**

We searched PubMed, PsycInfo, Web of Science, and the Cochrane Collaboration to identify studies published from 1975-2020 that: (1) examined contraceptive behaviour, (2) included measures of ITU and future contraceptive use, and (3) included at least one quantitative measure of association between ITU and actual use. The inclusion criteria were: 1) examined contraceptive behaviour (excluding condom use only), (2) included disaggregated integral measures of ITU contraceptives and later contraceptive use, (3) included at least one quantitative measure of the association between ITU contraceptives and actual contraceptive use, (4) study population was women of reproductive age, (5) were peer-reviewed, and (6) written in English.

**Results:**

10 prospective cohort studies met the inclusion criteria; these provided 28,749 person-years of data (N=10,925). Although we could pool the data for unadjusted odds ratios, a metanalysis was not possible. We calculated that 6 of the 10 studies indicated significant, increased, unadjusted odds of subsequent contraceptive use after reporting ITU. Of those, 3 study analyses reported significant, positive adjusted odds ratios for the relationship between intention to use and later contraceptive use across varying covariates. The range of confounding factors, particularly around sub-populations, points to the need for more research so that a meta-analysis can be done in the future.

**Conclusions:**

People’s self-reported ITU contraception has the potential to be a strong predictor of subsequent contraceptive use. Few studies directly examined the relationship between ITU and contraceptive uptake and recruitment was primarily pregnant or postpartum samples.

## Introduction

Understanding people’s desire to use modern contraception is critical to ensuring programs support people to achieve their reproductive needs and preferences. Since the 1970s ‘unmet need for contraception’ has been the main measure of demand for contraception, with some revisions along the way ^1– 3^. Unmet need is defined as the number or percentage of women currently married or in a union who are fecund and desire to either terminate, limit, or postpone childbearing but who are not currently using a contraceptive method ^4^. Unmet need has been misinterpreted as a desire to use contraception when it actually measures a person’s fertility intentions and then assumes because they are not using contraception that they have a “need” or want to use it ^5, 6^. However, people’s fertility desires may or may not lead them to desire contraception, and thus “unmet need” may not necessarily align with people’s desires to use contraception ^7– 10^. In addition to this misinterpretation, recent research has shown further limitations of unmet need: the calculations used for global estimates differ ^4, 8, 11, 12^ and the focus on women in unions miscategorises and excludes many women in other arrangements ^7, 11, 13– 18^.

Ilene Spiezer *et al.*, in considering how to better apply a human rights and reproductive rights lens, suggest we need to advance person-centred measures that better reflect people’s needs and preferences ^6^. As such, if we want to understand the relationship between intention and use, we need measures that actually ask women whether they desire or intend to use. Intention-to-use (ITU) contraception captures a person’s interest in using contraception in the future by directly asking people their preferences. This may better predict future contraceptive use and could potentially be a way to estimate programmatic gaps more accurately for those who face barriers ^14^. Though ITU has been collected since the 1970s, it has yet to receive the same attention as other key family planning metrics (e.g., unmet need, additional/new users) ^16, 19– 21^.

To test the potential scope of ITU as a more person-centred measure to support more responsive contraceptive programme, we first conducted a scoping review and found that scholars working on ITU suggest that contraceptive intentions as a proximate predictor of future contraceptive use merits further research ^5, 12, 15, 16, 22– 24^. The earlier scoping review included a wider range of evidence and identified 112 papers and their operationalizations of ITU; here we build off of that work to examine a subset of the studies where the data collection design and reporting was sufficient to be able to assess whether ongoing and continued measurement of ITU has the potential to accurately predict subsequent contraceptive use for those who desire it. The research protocol is registered in PROSPERO ^25^.

## Methods

### Search strategy

**Figure 1.**
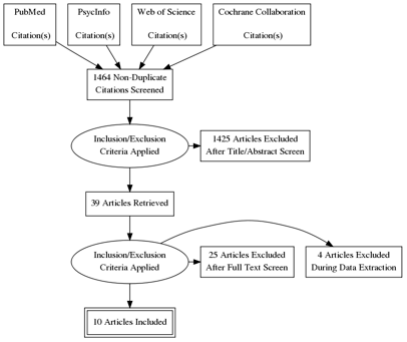
PRISMA.

The search strategy was informed by the earlier scoping review that examined the extent, range, and nature of the evidence on measuring ITU ^5^. This scoping review indicated that further analysis was needed to better understand whether ITU has significant effects on subsequent contraceptive uptake, so we performed a systematic review to examine this relationship. For this systematic review, we followed the PRISMA guidelines for reporting systematic reviews and meta-analyses ^26^. Please see Figure 1. We searched PubMed, PsycInfo, Web of Science, and the Cochrane Collaboration for studies published between 1975 and August 2020 using search terms relevant to intent-to-use and contraceptive use. The search terms and strategy are shown in the protocol ^25^.

### Inclusion and exclusion criteria

The study design included in the review were experimental, quasi-experimental, or observational studies with either a pre/post or treatment/control comparison. Studies were eligible for inclusion if they: (1) examined contraceptive behaviour (excluding condom use only), (2) included disaggregated integral measures of ITU contraceptives and later contraceptive use, (3) included at least one quantitative measure of the association between ITU contraceptives and actual contraceptive use, (4) the study population was women of reproductive age, (5) were peer-reviewed, and (6) were written in the English language. There were no limits to study inclusion based on the study setting. Studies were excluded if the full text was not accessible, not published in a journal (e.g., dissertations), or not written in English.

### Study selection and data extraction

We exported the search results into Endnote21 to remove duplicates and then imported the de-duplicated results into Excel 2021. Two authors (VB and SE) independently screened 1,464 titles and abstracts ^27^. Where discrepancies arose, the authors resolved disagreements through discussion between the reviewers. Subsequently, SE and VB independently reviewed 39 full-text articles to ascertain their eligibility for inclusion and resolved disagreements through discussion. Data extracted included the year of publication, study purpose, location, study design, sample size, participant characteristics, follow-up period in months, type of contraceptive used, measurement of ITU, measurement of contraceptive use, attrition, number of participants who reported ITU contraception who subsequently did and did not use contraception, the number of participants who reported no ITU contraception who then did and did not use contraception, and effect measure and size (See Table 1). Data were then independently extracted from the 10 included articles by one author (SE) using a predesigned data extraction form ^27^. One author (KW) reviewed the full papers and checked the data extraction. We calculated unadjusted odds ratios for the included studies, as several did not report adjusted odds ratios for the relationship between ITU and contraceptive use. We report both our calculations of the unadjusted odds ratios and author’s adjusted odds ratios with the variables adjusted for in our presented results.

**Table 1.** Description of included papers.

Study	Aim	Participant Sample Size at Baseline and Follow Up(s)	Study Location	Study Design	Follow up Period	Quality Rating	Effects Measure Reported in Study	Results	Calculated Unadjusted Odds Ratio (CI)	What Significance Test is Testing For	Measure of intention	Measure of contraceptive use
Curtis & Westoff 1996	To examine the relationship between stated ITU contraceptives and subsequent use during a three-year period	908 women married to same partner at both surveys, non- users at initial survey	Morocco	Longitudinal (cohort)	3 years	High (10)	Odds Ratio	OR: 6.78 *** aOR: 2.6 *** aOR (with interactions): 2.40	7.40 (5.51, 9.93)	Whether contraceptive use significantly increased among those reporting ITU compared to those not reporting	All ever-married respondents who weren’t using a contraceptive method were asked: “Do you intend to use a method to delay or avoid pregnancy at any time in the future/in the next 12 months?”	Not described
Lori *et al.* 2018	To examine the uptake and continuation of family planning following enrolment in group versus individual ANC	240 pregnant women at ANC settings at baseline and 164 at endline	Ghana	Longitudinal (cohort)	1 year	High (10)	Odds Ratio	aOR (any method): 1.549 aOR (any modern method):1.085	2.17 (1.11, 4.25)	Same as Curtis and Westhoff, 1996	Not described	Self-reported use
Sarnak *et al.* 2020	To assess the dynamic influence of unmet need on time to contraceptive uptake, as compared with that of contraceptive intentions and their concordance	747 sexually active, non-contracepting, fecund, women	Uganda	Longitudinal (cohort)	6,12,18, 24, and 36 months	High (9)	Hazard Ratio	HR: 1.65 * aHR: 1.45 *	3 years 4.48 (3.13, 6.42) 30 months 3.75 (2.62, 5.38) 24 months 3.22 (2.24, 4.62) 18 months 2.59 (1.79, 3.75) 12 months 2.27 (1.55, 3.33)	Same as Curtis and Westhoff, 1996	Non- contracepting women were asked whether they would use contraceptives in the future	Use of modern contraception
Tang *et al.* 2016	To (1) calculate the incidence of LARC use among postpartum Malawian women, and (2) assess if LARC knowledge and ITU LARC were associated with LARC uptake.	539 postpartum women (3 months), 480 (6 months), and 331 (12 months)	Malawi	Longitudinal (cohort)	3, 6, and 12 months after delivery	High (9)	Hazard Ratio	HR (implant use only): 1.88 ** aHR (implant use only): 1.95 *	1.05 (.67, 1.64)	Same as Curtis and Westhoff, 1996	Contraceptive methods she was planning to use in the first year after delivery	Self-reported use
Adelman *et al.* 2019	To evaluate which characteristics collected at the point of abortion are associated with contraceptive use over the extended postabortion period for women.	500 postabortion patients	Cambodia	Longitudinal (cohort)	4 and 12 months	Medium (7)	Odds Ratio	OR (4 months): 7.89 *** OR (12 months): 3.32 *** aOR (4 months): 4.60 *** aOR (12 months): 2.38	4.55 (3.00, 6.92)	Testing whether those who reported intention to use had different actual use compared to those who were undecided or reported they weren’t going to use a method	Not described	Self-reported use
Adler *et al.* 1990	To understand adolescent beliefs about contraception and their intention to use	325 postpartum, low-income, breastfeeding contraceptive initiators	USA	Longitudinal (cohort)	1 year	Medium (7)	Correlation coefficient	Pill (female): 0.42 *** Pill (male): 0.10 Diaphragm (female) 0.27 *** Diaphragm (male): 0.27 * Withdrawal (female): 0.20 ** Withdrawal (male): 0.46 ***	NA	Testing correlation of intention to use method with frequency of use in the following year	7-point scales responses to the statement "If I do have intercourse in the next year, I am ([very unlikely to very likely]) to ever use [method X] for birth control."	Self-reported use
Borges *et al.* 2018	To examine the effect of pregnancy planning status on the relationship between ITU and current use of contraceptives among postpartum women	474 ANC patients	Brazil	Longitudinal (cohort)	6 months after birth	Medium (6)	Concordance	28.9% concordance between contraceptive preference and subsequent contraceptive use.	1.48 (.54, 4.04)	Only assess significance by demographic or pregnancy planning group, not overall significance between ITU and contraceptive use	Women were asked while pregnant what type of contraceptive they intended to use after childbirth	Self-reported use and for those who reported more than one method, the most efficient was used.
Callahan & Becker 2014	To link women’s contraceptive uptake and experience of unwanted pregnancy between 2006 and 2009 to their unmet need status and their stated ITU contraceptives in 2006	3,933 married women at baseline and 3,687 at endline	Bangladesh	Longitudinal (cohort)	3 years	Medium (8)	Odds Ratio	OR (women with unmet need): 8.29 * OR (women with no unmet need): 7.17 *	7.25 (5.50, 9.56)	Same as Curtis and Westhoff, 1996	Pregnant and nonpregnant married women younger than 50 were asked: “Do you think you will use a method to delay or avoid pregnancy at any time in the future?” and were asked which method they intended to use	Self-reported use
Davidson & Jaccard 1979	To examine whether within versus across-subject procedures are more accurate for predicting behaviour from attitudes	279 married women at baseline and 244 at endline	USA	Longitudinal (cohort)	2 years	Medium (6)	Behavioural Intention B correlation	Correlation (for contraceptive use): 0.68 **	NA	Correlation between intention to use method and use within the next 2 years	7-point Likert scale measuring from likely to unlikely response to the statement: “I intend to use contraception within the next 2 years”	Self-reported use
Davidson & Morrison 1983	To understand factors that moderate the attitude-behaviour relation	221 married women, aged 18-38 years	USA	Longitudinal (cohort)	1 year	Medium (6)	Phi coefficients	Within and across subjects Condoms (within subjects): 0.86 ** Condoms (across subjects): 0.63 ** Pill (within subjects): 0.83 ** Pill (across subjects): 0.77 ** IUD: (within subjects): 0.94 ** IUD: (across subjects): 0.85 ** Diaphragm (within subjects): 0.92 ** Diaphragm (across subjects): 0.78 **	NA	Tests whether difference between within and across subject Phi-square coefficients is significant	Respondents intending to use a birth control method during the next year were asked what method they intended to use.	Self-reported use
Dhont *et al.* 2009	To investigate unmet need for LARCs and sterilization among HIV-positive pregnant women, and the impact of increased access to LARCs in the postpartum period on their contraceptive uptake	219 HIV-positive pregnant women at ANC settings at baseline and 205 at endline	Rwanda	Longitudinal (cohort)	9 months after birth	Medium (6)	Percentages	53% pregnant women reported an intention to use a LARC or to be sterilised after delivery 72% of women who had intended to start using a LARC actually did so at a site offering LARCs compared to only 4% of women at public FP sites ***	1.23 (.48, 3.21)	Tests whether LARC uptake at Site A (public FP services) were different than at Site B (guaranteed implant and IUD services)	Not described	Not described
Roy *et al.* 2003	To investigate women’s ITU a method as a measure of contraceptive demand	421 female participants in the 1992-92 National Family Health Survey	India	Longitudinal (cohort)	6 years	Medium (7)	Proportions	Of the 421 women who were asked the NFHS question on contraceptive intentions, 127 stated that they would use a method in the future. More than half (51%) of the women stating they would use a method in the future, did not do so during the intersurvey period compared to 29% of respondents who had said they would not practice family planning actually did so **	2.53 (1.53, 3.60)	Testing whether those who intended to use contraceptives were significantly more likely to use compared to those who had not planned on using a method	Not described	Self-reported use
Johnson *et al.* 2019	To understand how women’s prenatal infant feeding and contraception intentions were related to postpartum choices	223 postpartum women at baseline; 214 women postpartum in the hospital and 119 women at postpartum visit at <43 days	USA	Longitudinal (cohort)	Not specified	Low (5)	Correlation coefficient	Prenatal contraceptive intention and postpartum in-hospital correlation: 0.41 *** Prenatal contraceptive intention and postpartum visit choice correlation: 0.47 **	0.75 (.47, 1.22)	Correlation between prenatal contraceptive intention and in-hospital and postpartum visit method choice	Not described	For the analysis, contraceptive choice was characterized as no contraceptive method versus LARC

*p<.05, **p<.01, ***p<.001

### Assessment of risk of bias

One author (SE) assessed the risk of bias using the Joanna Briggs Institute Critical Appraisal Checklist for Cohort Studies ^28^, which assesses the trustworthiness, relevance and results of cohort studies. A scoring system assigns a score of 1 or 0 against each risk of bias domain. The scores were assigned and then summed across each domain, and studies were given a score ranging from 1 to 11. Subsequently, studies were classified into low (score below 5), medium (score of 6 to 8) and high quality (score above 8). Table 2 outlines the results of the assessment for each study.

**Table 2.** Summary of the findings from the included papers.

Study	Quality Rating	Calculated Unadjusted Odds Ratio (CI)	Author Reported Adjusted Odds Ratios (CI) for ITU coefficient on contraceptive use, and factors adjusted for	
Curtis & Westoff 1996	High (10)	7.40 *** (5.51-9.93)	2.64 *** (CI not given)	Categorical: fecundity, wanted last birth, fertility preference, prior contraceptive use, discussed family size with partner, attitudes about family planning messages in media, listened to radio weekly, education, residence, age, births, child deaths Continuous: number of living children *Note*: do not include results for interacted model
Roy *et al.* 2003	Medium (7)	2.53 *** (1.53-3.60)	Contraceptive use reported as regression outcome, intention to use not distinctive predictor variable but as a stratifier variable	
Dhont *et al.* 2009	Medium (6)	1.23 (0.48-3.21)	Contraceptive use not reported as regression outcome	
Callahan & Becker 2014	Medium (8)	7.25 *** (5.50-9.56)	Contraceptive use not reported as regression outcome	
Tang *et al.* 2016	High (9)	1.05 (0.67-1.64)	HR: 1.95 ** (1.28-2.98)	Age, parity, education, having a friend using the implant, HIV status, having trouble obtaining food, clothing, or medications
Borges *et al.* 2018	Medium (6)	1.48 (0.54-4.04)	Contraceptive use reported as regression outcome, intention to use not distinctive predictor variable	
Lori *et al.* 2018	High (10)	2.17 * (1.11-4.25)	*Note*: postpartum, modern method only 1.085 (0.444-2.655)	Age, gravida, religion, highest level of education
Adelman *et al.* 2019	Medium (7)	4.55 *** (3.00-6.92)	*Note*: ITU not presented in final adjusted models Outcome is 80% “continued contraception use” over 4 month: 7.98 *** (2.99-20.83) Note: outcome is 80% “continued contraception use” over 12 months: 3.32 ** (1.35-8.20)	Categorical: age, SES, residence, education, marital status, occupation, number of living children, number of previous abortions, abortion method, disclosure of abortion, previous contraception use, postabortion contraceptive intention, fertility intention, contraceptive decision making
Johnson *et al.* 2019	Low (5)	0.75 (0.47-1.22)	Contraceptive use not reported as regression outcome	
Sarnak *et al.* 2020	High (9)	36 months 4.48 *** (3.13-6.42)	36 months: 1.45 *** (1.22-1.73)	Categorical variables: age, parity, education, residence, wealth quintile

*p<.05 **p<.01 ***p<.001

### Data synthesis

Although some of the included papers did report relationships between intention to use and contraceptive use adjusted for a variety of covariates, these covariates are not the same across different studies. This means that either different studies included completely different covariates in their adjusted models or the way similar covariates were measured was not comparable across studies. Therefore, we calculated unadjusted odds ratios for the relationship between ITU and contraceptive use and reported on the adjusted ratios reported by authors. Despite the small sample size, we attempted to run a meta-analysis that combined the results of the studies for which we were able to calculate unadjusted odds ratios, as this would have generated a more robust source of evidence. However, meta-analysis diagnostics indicated that the high degree of variation across studies in follow up times, predictor and outcome measures, and sample populations (See Table 2) precluded pooling the data for a meta-analysis. This is the first attempt to systematically synthesise this information, and more studies that assess the longer-term relationship between reported intent to use and contraceptive use are needed for any future meta-analyses.

## Results

This is the first attempt to systematically synthesise this information, and more studies that assess the longer-term relationship between reported intent to use and contraceptive use are needed for any future meta-analyses (see Table 1).

### Study characteristics

The search yielded 1,464 articles. Many papers were excluded because they did not have a clear definition of intention to use (732), did not state an association between intention to use and contraceptive use (235), did not meet the study design requirements (238), did not contain sufficient information in the text to be assessed against the inclusion criteria (30), focused on condoms (161), did not include a measure of contraceptive use (61) or focused on only on the drivers of intention to use and did not test the association with actual use (17).

After the initial abstract screening and full paper review, a total of 10 articles were included ^27^. One of the 10 studies was conducted in the USA. The remaining studies were undertaken in low- and middle-income country (LMIC) settings: Bangladesh (n=1), Brazil (n=1), Cambodia (n=1), Ghana (n=1), India (n=1), Malawi (n=1), Morocco (n=1), Rwanda (n=1), and Uganda (n=1). All 10 studies were longitudinal cohort studies with pre-and post-tests or treatment and control groups. The characteristics of the studies, such as study aim, population, location, study design, follow up period, quality rating, effects measures, measure of ITU and measure of contraceptive use, are summarized in Table 1.

### Number and characteristics of participants

The number of participants varied between studies from 219 to 3,933, while six papers had sample sizes of approximately 200 to 300 participants. The papers looked at a variety of different participants – either women as broad category (e.g., sexually active or married) or at different points in their reproductive career (e.g., pre and post-partum). Two papers sampled married women ^16, 17^; two papers sampled postpartum women ^29, 30^; two papers sampled pregnant women ^31, 32^ and another two sampled sexually activity women ^7, 33^. Only one paper looked at women post-abortion ^34^. These papers provide 28,749 person-years of data (N=10,925).

### Definition of measures and outcomes

Half of the 10 included studies did not describe how exactly intention-to-use contraception was measured, and no details are provided on the exact wording of the items used to solicit information on the intention to use contraception ^29, 31– 34^. Of the remaining studies, three used items that asked about the intention to use contraception in the future with no exact time frame specified ^7, 16, 33^. Only one study used items that asked about intention to use contraception within a specific time; the time frame used was within the year ^30^.

In contrast, the majority of included studies did outline how they captured the outcome measure, contraceptive use. All of the studies used self-reported contraceptive use as the outcome measure (n=10). However, Johnson *et al.* used clinical records and two studies did not specify how they captured contraceptive use ^17, 29, 32^.

There was extensive heterogeneity in the measures used to report associations or effects in the included studies. Four papers used odds ratios to examine the relation between intention-to-use and use of contraception ^7, 16, 31, 35^. Across the studies that used odds ratios, researchers compared women who intended to use contraception to women who did not intend to use any method. These four studies found higher odds of women using contraception if they had planned to use it previously; this finding was statistically significant at p<.001 for three of the four studies. One paper used correlation coefficients ^29^, and two papers used hazard ratios ^7, 30^. The remaining papers reported on their findings using “concordance” ^33^, and simple percentages or proportions ^32, 34^.

### Associations

Of the 10 studies for which we calculated unadjusted odds ratios of contraceptive use by intention to use status, six had significant, increased odds of subsequent contraceptive use after reporting an intention to do so at an earlier point, see Table 2. The unadjusted associations range from 0.75–7.40 based on odds ratios. Of the 10 included studies, five reported on an adjusted relationship between intent to use as a predictor variable and contraceptive use as an outcome variable. Of these, four found significantly increased odds or hazards of contraceptive use given stated intent to use at the initial measurement. These studies adjusted for a variety of covariates, with the most common being age, measures of the number of pregnancies, and education. As would be expected, the magnitude of significant unadjusted odds ratios generally decreases with adjustment for covariates, however the strength of the association does not. In one case, Tang *et al.* (2016), our unadjusted odds ratio was non-significant, while the author’s calculation of an adjusted hazard ratio was. In the study conducted by Lori *et al.* (2018), our unadjusted calculation was significant at the p<.05 level while the authors’ adjusted calculation is non-significant.

### Specific contraceptive methods

Two of the included papers examined only long acting reversible method (LARC) use at follow up ^30, 32^. Three studies included only what would be considered modern contraceptive methods, including LARCS such as IUDs and implants, and shorter term methods like pills, injectables, vaginal rings, and condoms, alongside sterilization ^29, 33, 35^. The remaining studies grouped contraceptive methods into various groupings, such as ‘modern’, ‘modern and reversible’, ‘modern and permanent’, and ‘traditional’ ^7, 16, 17, 31, 34^.

### Time frame

There were also significant differences in the intervals between baseline and follow-up within the included studies. Most of the studies examined the relationship between intention to use and contraceptive use over long-term (longer than one-year) periods, ranging from one-year follow up measurements to six years in between measurements ^7, 16, 17, 31, 34, 35^. Some of these studies of longer duration included intervening measurements at specified month-intervals ^7, 30, 35^. The differences in odds ratios of contraceptive use at these intervals especially highlights the need for subsequent work to focus on specific intervals to better understand the duration range of intention to use reports. The remaining papers examined contraceptive use for less than one year, or the duration of follow up was unspecified ^29, 32, 33^.

### Population

Of the 10 studies included, six focused in and around pregnancy; this refers to the antenatal, postabortion, and postpartum period. Two of the 10 studies examined intention to use contraception among women in the postpartum period and followed up on whether women’s intention had transformed into use over the following 12 months ^4, 29, 30^. A further three studies examined women’s choice to use contraception in the antenatal period and followed up six months to one year after to see if they were using a method ^31– 33^.

Only one study looked at the intention to use among women following an abortion ^35^. In Cambodia, Adelman *et al.,* examined what characteristics collected at the point of abortion are associated with oral contraceptive use at four and 12 months after the abortion. Intention to use contraception was found to be positively associated with increased contraceptive use over the year ^35^.

 The remaining four studies looked at the intention to use contraception among women with partners, including married women ^7, 16, 17, 34^. Using longitudinal data from rural Bangladeshi women (n=2,500), Callahan and Becker found that intention to use a method was predictive of subsequent contraceptive use for women with and without an unmet need. Only two of these studies specified whether the women were non-users ^7, 16, 17^. In Uganda, Sarnak *et al.,* compared unmet need and contraceptive adoption to contraceptive intentions and use ^7^. They found that women who intended to use contraception in the future used contraceptives significantly earlier (aHR = 1.45, 95% CI = 1.22-1.73) than those who did not intend to use contraception ^7^. Interestingly, women with an intention to use but not classed as having no unmet need had the highest rate of adoption compared to those with no unmet need and no intention to use (aHR = 2.78, 95% CI = 1.48-5.258 ^6^. The follow-up period to see if married women’s intentions had turned into actual contraceptive use was a one-to-three-year period in this set of studies ^7, 16, 17, 34^.

### Quality of evidence in included studies

We used the Joanna Briggs Institute Critical Appraisal Checklist for Cohort Studies ^28^, which assesses the trustworthiness, relevance and results of cohort studies, to rate the quality of each study using the following domains: the sample, exposure measures, confounding factors, outcome measures, follow-up time reported, and type of analysis used. Four studies were graded as high quality, and five were of medium quality. One study was classed as low quality.

## Discussion

In this review, we found that there are significant positive associations between intention to use a contraceptive method and actual use in six medium- to high-quality studies. Yet the heterogeneity across the papers poses an analytical challenge for us to be able to really interrogate the potential of this person-centred measure; this in itself is a finding and speaks to the need for (1) refining the outcomes to measure intention to use, and (2) identifying a) which relevant variables need to be included in adjusted models and b) how these variables can be measured in ways so that they are comparably reported across studies.

### Refining the outcomes

Reading across the papers, there is inconsistency in how ITU is currently operationalized and applied. This analysis found that five (n=5) papers did not provide details on the wording of the items used to measure ITU ^29, 32– 35^. Based on what information is available from the included papers, five (n=5) papers captured goal intentions ^7, 16, 17, 24, 35^ whereas four (n=4) captured implementation intention ^23, 30, 31, 34^. This finding is significant because established behavioural theory suggests that distinguishing the type of intention may be helpful as implementation intentions are more likely to translate into the behaviour than goal intentions ^36^. Gollwitzer and Sheeran helpfully distinguish between goal intention and what people plan to do some time in the future ^37^. In contrast, implementation intentions are more specific regarding when, where, and how one's achievement of an intention will occur. Implementation intentions tend to be oriented towards a particular action, whereas goal intentions tend to be outcomes achieved by performing several actions ^37^. Gollwitzer and Sheeran argue that goal intentions do not prepare people for dealing with the problems they face in initiating, maintaining, disengaging from, or overextending themselves in realizing their intentions ^37^. In contrast, an implementation intention sets out the when, where, and how in advance and is a form of planning that bridges the intention-behaviour gap, increasing the likelihood of intentions being realized ^37^. Unfortunately, none of the papers included distinguished between goal and implementation intentions. Additional research on how ITU is conceptualized and operationalized is needed to understand how different types of intentions (e.g., goal vs implementation) predict contraceptive use and continuation. To address this, further research in needed using standardized ITU and outcome measures and similar follow-up durations amongst similar populations to assess the magnitude and direction of associations between ITU and contraceptive use.

### Adjusting for confounders

Given the heterogeneity, several potential confounding variables could affect whether an intention to use contraception leads to future contraceptive use. These possible confounding variables make it difficult to establish a causal link between ITU and contraceptive use. This review points to several potential confounding variables to consider in future work.

Several studies in this review focused on populations during and around pregnancy. This could be an artefact of research study design as recruiting women attending pregnancy-related services may be easier. It could be an artefact of programme design in that women are more likely to engage in healthcare during pregnancy. Similarly, parity and relationship status may also affect whether an intention to use contraception translates into actual use. Future research should examine how pregnancy status may affect intentions to use contraception compared to women seeking to prevent pregnancy who are not pregnant.

Another variable that may affect the relationship between intention to use and actual use is the type of contraception method being considered. For example, long-acting reversible contraceptive methods may require more commitment and planning, whereas short-acting methods may be easier to access and use. Hence, the specific type of method may differentially affect the ease or difficulty of a person transforming their intentions into action. Work on developing a psychometric scale on contraceptive intent highlighted that contraceptives are a form of medication, and the woman's desire and adherence to them are influenced by beliefs about the medicine ^10^. Another variable we noted is how long it may take to move from intention to action and when to measure if this execution has taken place. Several studies reported different follow-up durations ^7, 30, 35^. Our findings are too inconsistent in reporting the timeframe to make any generalizations about the appropriate time to move intention to action; the literature on behaviour implementation suggests that this is an important avenue for future study.

The range of potential interceding factors that emerged in the review point to the fact that contraceptive behaviour is a complex psychosocial process shaped by the confluence of individual and contextual factors ^10^. Such factors may help explain how pregnancy and relationship status are related to intentions or use of specific methods, whether goal or implementation intentions result in actual use, and over what timeframe intentions to use contraception are likely to transform into action. In turn, this can contribute to better understand people’s needs and preferences and how we can align programs to support them to achieve their reproductive goals and contraceptive goals.

There are several limitations to this review. There were relatively few studies that met the inclusion criteria. The relationship between ITU and contraceptive uptake was not the primary outcome of interest for those included papers. Thus, we had to calculate an odds ratio to estimate that relationship. Therefore, we treat our results as indicative. Another limitation is that the samples recruited for the included studies were primarily pregnant or postpartum samples—the desire to start sexual activity and contraception may be different for these populations compared to others. Geographic settings, particularly the difference in health systems and contraceptive access, may also explain the differences we found. In addition, other factors (e.g., cultural and social norms, knowledge about contraceptive methods, personal beliefs) may all contribute to reproductive and contraceptive intentions, decision-making, and subsequent use, and require further consideration.

## Conclusion

Six studies indicated significant, increased odds of subsequent contraceptive use after reporting ITU and show a significant positive association between desire to use contraception and actual use. This suggests that self-reported ITU contraception may be a strong predictor of subsequent contraceptive use and a promising alternative measure of demand for contraception. As a person-centred measure, we need further high-quality research that measures the relationship between intent-to-use and contraceptive use using standardized measures and more fully considering the range of additional factors that may influence both ITU and subsequent use.

## Data Availability

OSF: Toward person-centred measures of contraceptive demand: a systematic review of the intentions to use contraception and actual use. https://doi.org/10.17605/OSF.IO/6FXQT ^27^. The project contains the following underlying data: ITU Sys Review underlaying data citations (data citations for the systematic review). ITU Sys Review underlaying data citations screening too (screening tool). ITU Sys Review underlaying full papers (list of full papers for the systematic review). ITU Sys Review underlaying full paper screening tool (screening tool for full papers for the systematic review). OSF: Toward person-centred measures of contraceptive demand: a systematic review of the intentions to use contraception and actual use. https://doi.org/10.17605/OSF.IO/6FXQT ^27^. This project contains the following extended data: Supplementary Table 1. (Description of included studies) Supplementary Figure 1. (PRISMA flowchart) Data collection tool. (raw data used in analysis) OSF: PRISMA and PRISMA for abstracts checklists for ‘Toward person-centred measures of contraceptive demand: a systematic review of the intentions to use contraception and actual use’. https://doi.org/10.17605/OSF.IO/6FXQT ^27^. Data are available under the terms of the Creative Commons Zero "No rights reserved" data waiver (CC0 1.0 Public domain dedication).
