# Supplementary material for: Toward person-centred measures of contraceptive demand: a systematic review of the relationship between intentions to use and actual use of contraception
Source: Gates Open Res. 2025 Feb 28;8:1. Originally published 2024 Jan 3. [Version 3] doi: 10.12688/gatesopenres.15078.3 (PMC11634881; doi:10.12688/gatesopenres.15078.3)
Supplement: Peer review history

# Reviewer report: Reviewer response for version 3

**DOI:** 10.21956/gatesopenres.17707.r39461

**Published:** 2025-03-03

**Authors:** Emily R Boniface

**Affiliations:** 1 Health Systems & Policy, OHSU-PSU School of Public Health, Portland, Oregon, USA; 2 Oregon Health & Science University, Portland, Oregon, USA

The authors have successfully addressed all my previous comments.

Are the rationale for, and objectives of, the Systematic Review clearly stated?

Yes

Is the statistical analysis and its interpretation appropriate?

Yes

Are sufficient details of the methods and analysis provided to allow replication by others?

Partly

Are the conclusions drawn adequately supported by the results presented in the review?

Partly

Reviewer Expertise:

Biostatistics, contraception use, person-centered contraceptive care

I confirm that I have read this submission and believe that I have an appropriate level of expertise to confirm that it is of an acceptable scientific standard.

# Reviewer report: Reviewer response for version 2

**DOI:** 10.21956/gatesopenres.17363.r38605

**Published:** 2024-12-11

**Authors:** Jean François Régis Sindayihebura

**Affiliations:** 1 University of Burundi, Bujumbura, Burundi

**ABSTRACT**

 The authors have found that the measure of the effect of the demand for family planning methods on the actual use of contraception has always been operationalized by Unmet Need. However, the latter can also characterize women who have no intention of using it, and therefore don't really participate in the expression of demand for FP methods (unmet needs include in the analysis people who didn't want the recent birth/pregnancy in progress, or who would not like to get pregnant in the future, without having the desire to use contraception). Thus, they would like to focus the analysis on the actual needs expressed by the intention to predict contraceptive use.

 By carrying out a systematic review of previous studies, they selected, according to rigorous inclusion and selection criteria, 10 studies from which they show that intention to use contraception defines its actual use in the future. The conclusion is drawn from the results of calculating unadjusted odds ratios, as the control variables were not common to the all selected studies.

**ANSWERS TO QUESTIONS**

***Are the rationale and objectives of the systematic review clearly stated?***

 The researchers justify their study of the impact of intention to use contraception on subsequent actual use by contrasting it with studies of unmet need for FP. According to these authors, unmet need is not sufficient as a measure of the desire to use contraception (unmet need includes people who did not want the recent birth/pregnancy or who would not like to become pregnant, without having the desire to use contraception). In this way, they intend to focus the measure on people who actually express the need for contraception, in order to assess its effect on actual use.

 However, it seems that a systematic review of the literature is not enough to achieve this objective. The reviewed works are the ones that best meet this objective. They simply justify why these analyzed studies were carried out, without being able to justify their own (this systematic review).

 The authors reviewed a multitude of papers, from which they eventually retained only 10. They have the advantage of formulating a good problematic than that of the study focused on people in need of contraceptives (i.e. those who express the intention to use contraception).

***Are the methods and analysis detailed enough to be replicated by others?***

 The authors describe at length the objective process of inclusion and selection of the studies reviewed. They also cite the calculation of unadjusted odds ratios because the variables used to control for the effect of intention to use contraception on actual contraceptive use were not the same in the reviewed studies. However, no methodological approach to calculating these unadjusted odds ratios is mentioned to allow reproducibility. Mathematical details of the methodology are needed.

 The reader will also wonder how this calculation was possible when no manipulation of the databases is mentioned. What was the target population, or what were the statistical units? 

***Are the statistical analysis and its interpretation appropriate?***

 The statistical analysis and its interpretation are appropriate, but the lack of detail in the methodology expressed above prevents us from drawing any objective conclusions.

***Are the conclusions drawn adequately supported by the results presented in the review?***

 The results presented appear to be a continuation of the systematic review, the selection of studies to be included and the methodology. The reader will find that the results present in unclear and immense content the effect of intention to use contraception on its actual use. The authors would do well to be brief and concise.

 The conclusion presents the essential results, but lacks the necessary elements. A reminder of the context, objective and methodological approach. It should also present the strengths and limitations.

 The conclusion is therefore relatively short.

Are the rationale for, and objectives of, the Systematic Review clearly stated?

Partly

Is the statistical analysis and its interpretation appropriate?

Partly

Are sufficient details of the methods and analysis provided to allow replication by others?

No

Are the conclusions drawn adequately supported by the results presented in the review?

Yes

Reviewer Expertise:

Demography ; Human geography ; Social Sciences

I confirm that I have read this submission and believe that I have an appropriate level of expertise to confirm that it is of an acceptable scientific standard, however I have significant reservations, as outlined above.

# Response

**Published:** 2025-02-13

**Authors:** victoria boydell

Thank you so much for your feedback on version 1 of the paper. We have made substantial revisions to the paper based on the input of the two other reviewers and many of these changes address the issues you raised. Your concerns about the framing of the paper have been addressed. We appreciate the input and think it will make for a much-improved paper.

# Reviewer report: Reviewer response for version 2

**DOI:** 10.21956/gatesopenres.17363.r37271

**Published:** 2024-07-17

**Authors:** Anastasia J Gage

**Affiliations:** 1 Department of International Health and Sustainable Development, School of Public Health and Tropical Medicine, Tulane University, New Orleans, Louisiana, USA

The authors have adequately addressed the reviewers' comments. Table 1 now specifies the study designs, study populations, sample size and other study characteristics, which is helpful. The authors have provided a justification for the inclusion of a "low-quality" study in the systematic review and now have a more thorough discussion of the study's limitations.

Are the rationale for, and objectives of, the Systematic Review clearly stated?

Yes

Is the statistical analysis and its interpretation appropriate?

I cannot comment. A qualified statistician is required.

Are sufficient details of the methods and analysis provided to allow replication by others?

Yes

Are the conclusions drawn adequately supported by the results presented in the review?

Yes

Reviewer Expertise:

Family planning, reproductive and maternal health

I confirm that I have read this submission and believe that I have an appropriate level of expertise to confirm that it is of an acceptable scientific standard.

# Reviewer report: Reviewer response for version 2

**DOI:** 10.21956/gatesopenres.17363.r37272

**Published:** 2024-07-10

**Authors:** Emily R Boniface

**Affiliations:** 1 Health Systems & Policy, OHSU-PSU School of Public Health, Portland, Oregon, USA; 2 Oregon Health & Science University, Portland, Oregon, USA

Thank you for your work on this important topic and the revisions, particularly the inclusion of Table 1, which have improved the clarity of the manuscript. I have a few additional suggestions to improve the interpretability of the study results.

Introduction:

- 2nd paragraph: the second sentence appears to be missing some words.
- 3rd paragraph, 1st sentence: "programme" should be "programmes".

Table 1:

- ANC abbreviation should be defined.

Results:

- Associations section: I appreciate the clarification regarding magnitude or odds ratios and strength of association in the response from authors. However, without any edits to the manuscript, the text as written remains unclear from a statistical perspective. I strongly suggest specifying that "strength of association" refers to the precision of the estimates, as that is not a typical use of the term and could easily be misinterpreted.
- Specific contraceptive methods: It would be very helpful to the reader to include these details in Table 1.
- There is a lot of redundancy between the "Number and characteristics of participants" and "Population" sections. Clarity would be significantly improved by combining them or removing duplicate information.

Are the rationale for, and objectives of, the Systematic Review clearly stated?

Yes

Is the statistical analysis and its interpretation appropriate?

Yes

Are sufficient details of the methods and analysis provided to allow replication by others?

Partly

Are the conclusions drawn adequately supported by the results presented in the review?

Partly

Reviewer Expertise:

Biostatistics, contraception use, person-centered contraceptive care

I confirm that I have read this submission and believe that I have an appropriate level of expertise to confirm that it is of an acceptable scientific standard, however I have significant reservations, as outlined above.

# Response

**Published:** 2025-02-13

**Authors:** victoria boydell

Thank you so much for your feedback on version 2 of the paper. We have reviewed all your comments and have incorporated all the suggested changes. We appreciate the input and think it will make for a much-improved paper.

# Reviewer report: Reviewer response for version 1

**DOI:** 10.21956/gatesopenres.16413.r36249

**Published:** 2024-04-16

**Authors:** Anastasia J Gage

**Affiliations:** 1 Department of International Health and Sustainable Development, School of Public Health and Tropical Medicine, Tulane University, New Orleans, Louisiana, USA

The extent to which contraceptive intention translates into actual contraceptive use has long been a subject of debate, even though studies generally show a positive correlation between intention and behavior. The research question in the present systematic review was straightforward. The authors examined whether contraceptive intentions predict subsequent contraceptive use. The inclusion and exclusion criteria were clearly specified. While all studies included in the review were longitudinal, not all employed an experimental or quasi-experimental design. Some included studies were observational. My comments on the present study are outlined below.

- The literature search strategy was comprehensive. Search terms were provided in PROSPERO and included the following: ((intent* OR intend*) AND (“to use”)) OR (intent* OR intend* OR willingness) AND (contracept* OR “birth control” OR “family planning”).  The literature was independently screened by two of the authors to determine the eligibility of studies for inclusion in the systematic review, and full text articles were independently reviewed by the same authors.
- While data were independently extracted from the 10 included articles by one author using a predesigned data extraction form, a second author reviewed the full papers and checked the data extraction.
- Of 39 articles that were retrieved, 25 articles were excluded after full text screen and 4 articles during data extraction, Unfortunately, the review authors did not fully account for the excluded articles. It would be instructive to know the likely impact of their exclusion on the conclusions of the systematic review.
- One consideration was the extent to which the authors described the studies in adequate detail. Although Table 1 does not provide details about research designs, study populations, interventions (if applicable), and study settings, this information is described in the text and gives insights into variations in the study populations and study settings.
- As all 10 studies included in the present systematic review were longitudinal cohort studies (with pre-and post-tests or treatment and control/comparison groups), the authors used the Joanna Briggs Institute Critical Appraisal Checklist for Cohort Studies to assess risk of bias. One of the included studies had a “low quality” rating and was retained in the systematic review. Could the authors kindly justify its retention? If this study were to be omitted, what would be the implications for the interpretation of the results of the review?
- The authors provided a satisfactory discussion of observed heterogeneity in the results of the review. They reported heterogeneity in (a) measures used to report associations or effects in the included studies (odds ratios, hazard ratios, correlation coefficients, simple percentages/proportions); (b) study design (which included non-randomization); (c) analysis (non-adjustment or adjustment for covariates).
- In the discussion section, the authors highlighted the importance of adjusting for possible confounding variables, such as parity, relationship status, type of contraceptive method. Contraceptive decision making is also shaped by factors that were not mentioned, including cultural and social norms, knowledge about contraceptive methods, personal beliefs, and access to and supply of contraceptive methods. It is important to mention these factors when discussing the limitations of the study.
- The preceding comment (i.e., the importance of adjusting for confounding variables) begs the question as to whether the five studies that did not report an adjusted relationship between intent to use (predictor variable) and contraceptive use (outcome variable) should be included in the systematic review. I believe that these studies should not be included as they detract from the robustness of the results.

Overall, the present systematic review highlights (a) research gaps, (b) the need for standardized measures of intention to use contraception, and (c) the importance of distinguishing between goal intentions and implementation intentions when predicting subsequent contraceptive use, after adjusting for confounding variables.

Are the rationale for, and objectives of, the Systematic Review clearly stated?

Yes

Is the statistical analysis and its interpretation appropriate?

I cannot comment. A qualified statistician is required.

Are sufficient details of the methods and analysis provided to allow replication by others?

Yes

Are the conclusions drawn adequately supported by the results presented in the review?

Yes

Reviewer Expertise:

Family planning, reproductive and maternal health

I confirm that I have read this submission and believe that I have an appropriate level of expertise to confirm that it is of an acceptable scientific standard, however I have significant reservations, as outlined above.

# Response

**Published:** 2024-06-10

**Authors:** victoria boydell

The extent to which contraceptive intention translates into actual contraceptive use has long been a subject of debate, even though studies generally show a positive correlation between intention and behavior. The research question in the present systematic review was straightforward. The authors examined whether contraceptive intentions predict subsequent contraceptive use. The inclusion and exclusion criteria were clearly specified. While all studies included in the review were longitudinal, not all employed an experimental or quasi-experimental design. Some included studies were observational. My comments on the present study are outlined below. 

 The literature search strategy was comprehensive. Search terms were provided in PROSPERO and included the following: ((intent* OR intend*) AND (“to use”)) OR (intent* OR intend* OR willingness) AND (contracept* OR “birth control” OR “family planning”).  The literature was independently screened by two of the authors to determine the eligibility of studies for inclusion in the systematic review, and full text articles were independently reviewed by the same authors.

 While data were independently extracted from the 10 included articles by one author using a predesigned data extraction form, a second author reviewed the full papers and checked the data extraction.

 Of 39 articles that were retrieved, 25 articles were excluded after full text screen and 4 articles during data extraction, Unfortunately, the review authors did not fully account for the excluded articles. It would be instructive to know the likely impact of their exclusion on the conclusions of the systematic review.

***Response:** We have now added in this information.*

 One consideration was the extent to which the authors described the studies in adequate detail. Although Table 1 does not provide details about research designs, study populations, interventions (if applicable), and study settings, this information is described in the text and gives insights into variations in the study populations and study settings.

***Response:** Table 1, which was mistakenly excluded, does provide this information.*

 As all 10 studies included in the present systematic review were longitudinal cohort studies (with pre-and post-tests or treatment and control/comparison groups), the authors used the Joanna Briggs Institute Critical Appraisal Checklist for Cohort Studies to assess risk of bias. One of the included studies had a “low quality” rating and was retained in the systematic review. Could the authors kindly justify its retention? If this study were to be omitted, what would be the implications for the interpretation of the results of the review?

***Response: **We review a low-quality study in the systematic review because it meets all of the a priori inclusion requirements, which is part of the process of systematic reviews. It was post-hoc given a quality rating as part of a typical quality review for a study like this. We are happy to add this text regarding systematic review processes if it would aid in clarifying this for readers. If this study was removed, the existing findings would remain the same, we would just be removing one study with non-significant findings. It doesn't change the overall interpretation of the review, which is that there is not sufficient evidence to do a meta-analysis of ITU and further research should be conducted that would allow researchers to identify whether this is a successful and potentially more person-centered measure of contraceptive use.*

 The authors provided a satisfactory discussion of observed heterogeneity in the results of the review. They reported heterogeneity in (a) measures used to report associations or effects in the included studies (odds ratios, hazard ratios, correlation coefficients, simple percentages/proportions); (b) study design (which included non-randomization); (c) analysis (non-adjustment or adjustment for covariates).

 In the discussion section, the authors highlighted the importance of adjusting for possible confounding variables, such as parity, relationship status, type of contraceptive method. Contraceptive decision making is also shaped by factors that were not mentioned, including cultural and social norms, knowledge about contraceptive methods, personal beliefs, and access to and supply of contraceptive methods. It is important to mention these factors when discussing the limitations of the study.

***Response: **Thank you for the observation and we have made the change:* “ *In addition, other factors (e.g., cultural and social norms, knowledge about contraceptive methods, personal beliefs) may all contribute to reproductive and contraceptive intentions, decision-making, and subsequent use, and require further consideration.”*

 The preceding comment (i.e., the importance of adjusting for confounding variables) begs the question as to whether the five studies that did not report an adjusted relationship between intent to use (predictor variable) and contraceptive use (outcome variable) should be included in the systematic review. I believe that these studies should not be included as they detract from the robustness of the results.

***Response: **We have included papers that did not report the adjusted relationship between the predictor and the outcome variable to ensure thoroughness in our analysis and avoid introducing bias.*

 Overall, the present systematic review highlights (a) research gaps, (b) the need for standardized measures of intention to use contraception, and (c) the importance of distinguishing between goal intentions and implementation intentions when predicting subsequent contraceptive use, after adjusting for confounding variables.

# Reviewer report: Reviewer response for version 1

**DOI:** 10.21956/gatesopenres.16413.r36251

**Published:** 2024-04-16

**Authors:** Emily R Boniface

**Affiliations:** 1 Health Systems & Policy, OHSU-PSU School of Public Health, Portland, Oregon, USA; 2 Oregon Health & Science University, Portland, Oregon, USA

**Overall Comments: **This systematic review attempts to examine how well intention to use (ITU) contraception predicts future contraceptive use. The goal of identifying a more person-centered measure of desire for contraception is an important one. Unfortunately, the small number of studies identified in the analysis and the broad range of associations don’t support a claim that ITU is a better measure, and the authors rightly point out the clear need for more research.

**Abstract**

- Background: suggest removing the last portion of the final sentence. The study never actually compares ITU’s predictive ability to that of unmet need and I’d argue that the results don’t allow for a definitive conclusion about how well ITU successfully predicts future use.
- It’s a bit confusing to address study populations in the conclusion when they are not mentioned anywhere else in the abstract. Suggest including some mention of them in the results if they are an important part of the conclusion.

**Introduction**

- 1 ^st^ paragraph: the connection between understanding desire for contraception and access to a contraceptive program is unclear, as is the last sentence. Is the argument that people shouldn’t be classified as having unmet need given that they state they intend to use contraception in the future but are not currently using a method? I would think that would be a more reasonable assumption than categorizing someone as having unmet need who states that they do *not* intend to use a method in the future.
- 3 ^rd^ paragraph: “programme” should be “programmes”. Clarify that the scoping review was conducted previously; as currently written, the statement about the scoping review could be interpreted as referring to the current study.
- It would be helpful to clarify why the scoping review included so many more studies than the systematic review
- Suggest considering PMID 36841972 is part of the background literature.

**Methods**

- Please include the search terms used to identify papers to facilitate reproducibility
- Curious about the choice to exclude studies that looked at condoms given the fact that they were included as a method choice in several of the included studies, and in fact, some of the included studies even included “traditional” methods. I don’t necessarily have an issue with it, but some justification would be appreciated.

**Results**

- Data synthesis: the last sentence should be in the discussion rather than methods
- It would be helpful to include details on the reasons/n’s for the 1425, 25, and 4 studies that were excluded after applying the inclusion/exclusion criteria at various steps. I’m a little surprised by how few studies were ultimately included and it would be useful to see why others were excluded.
- The text states that study aim, population, location, study design, and follow-up period are included in Table 1, however, this is not the case. Please include columns for each of these variables, as well as sample sizes and titles. It would also be nice to be able to see the definition of methods used in each study. Perhaps dividing the information into 2 tables would be useful.
- Suggest including full list of papers as supplemental material.
- Without being able to see the sample sizes and follow-up periods for each paper, the statement about person-years of data is unclear. I’m assuming the statement implies just a few months of follow-up for almost 5,000 study participants. Is that correct? If not, more clarification is needed.
- Associations section: the distinction between magnitude and strength of association doesn’t have a statistical meaning, so it’s unclear what is being communicated at the end of this paragraph. Why doesn’t the strength of association change after adjustment if there are examples of significant unadjusted OR and non-significant aOR *and* vice versa? Please clarify.

**Discussion**

- Appreciate the nuance about goal and implementation intentions. Is that a distinction that was recognized by any of the excluded studies that did not assess future contraception use following report of ITU?
- Another limitation is the variability of geographic settings for the included studies. The analysis seems to assume that the relationship between ITU and subsequent method use is generalizable across settings, which is a fairly strong assumption given the possible differences in health systems and contraceptive access. Could the wide range of ORs be explained by some of these differences?
- It would be helpful to address/compare the results to unmet need. If the motivation for this study is that unmet need is an inadequate measure for predicting contraception use, how does this study compare and what do the results add? It doesn’t appear that there is currently enough evidence to support the claim that ITU is a better predictor of future use, so the conclusion seems to overstate it’s strength as a predictive measure.

Are the rationale for, and objectives of, the Systematic Review clearly stated?

Yes

Is the statistical analysis and its interpretation appropriate?

Yes

Are sufficient details of the methods and analysis provided to allow replication by others?

Partly

Are the conclusions drawn adequately supported by the results presented in the review?

Partly

Reviewer Expertise:

Biostatistics, contraception use, person-centered contraceptive care

I confirm that I have read this submission and believe that I have an appropriate level of expertise to confirm that it is of an acceptable scientific standard, however I have significant reservations, as outlined above.

## References

[1] : Assessing the Suitability of Unmet Need as a Proxy for Access to Contraception and Desire to Use It. *Stud Fam Plann* .2023;54(1) : 10.1111/sifp.12233 231-250 10.1111/sifp.12233 36841972 PMC10257191

# Response

**Published:** 2024-06-10

**Authors:** victoria boydell

**Overall Comments: **This systematic review attempts to examine how well intention to use (ITU) contraception predicts future contraceptive use. The goal of identifying a more person-centered measure of desire for contraception is an important one. Unfortunately, the small number of studies identified in the analysis and the broad range of associations don’t support a claim that ITU is a better measure, and the authors rightly point out the clear need for more research.

**Abstract.**

 Background: suggest removing the last portion of the final sentence. The study never actually compares ITU’s predictive ability to that of unmet need and I’d argue that the results don’t allow for a definitive conclusion about how well ITU successfully predicts future use.

***Response: **We have changed the wording to be more appropriate to the content of the paper and it now reads: “This systematic review examines the relationship between relationship between intentions to use and actual use of contraception and could potentially in developing responsive programs.”*

 It’s a bit confusing to address study populations in the conclusion when they are not mentioned anywhere else in the abstract. Suggest including some mention of them in the results if they are an important part of the conclusion.

***Response:** Noted and we have now included a mention of population in the conclusion of the abstract, which reads “The range of possible confounding factors, particularly around the different populations, points to the need for more research so that a meta-analysis can be done in the future.”*

**Introduction**

 1 ^st^ paragraph: the connection between understanding desire for contraception and access to a contraceptive program is unclear, as is the last sentence. Is the argument that people shouldn’t be classified as having unmet need given that they state they intend to use contraception in the future but are not currently using a method? I would think that would be a more reasonable assumption than categorizing someone as having unmet need who states that they do *not* intend to use a method in the future.

***Response: **Thank you for the observation and we have made the change and we have removed this statement to prevent confusion.*

 3 ^rd^ paragraph: “programme” should be “programmes”.

***Response:** Change has been made.*

 Clarify that the scoping review was conducted previously; as currently written, the statement about the scoping review could be interpreted as referring to the current study.

***Response: **Thank you for the observation and we have made the change to distinguish this and the earlier review: “The scoping review included a wider range of evidence and identified 112 papers and their operationalizations of ITU; here we build off of that work to examine a subset of the studies where the data collection design and reporting was sufficient to be able to assess whether ongoing and continued measurement of ITU has the potential to accurately predict subsequent contraceptive use for those who desire it.”*

 It would be helpful to clarify why the scoping review included so many more studies than the systematic review.

***Response:** Thank you for the observation and we have made the change: “The scoping review included a wider range of evidence and identified 112 papers and their operationalizations of ITU; here we build off of that work to examine a subset of the studies where the data collection design and reporting was sufficient to be able to assess whether ongoing and continued measurement of ITU has the potential to accurately predict subsequent contraceptive use for those who desire it.”*

 Suggest considering PMID 36841972 is part of the background literature.

***Response: **Thank you for the observation and we have made the change. This has been included in the references and citations.*

**Methods**

 Please include the search terms used to identify papers to facilitate reproducibility

***Response:** Thank you, we already direct the readers to the protocol – explicitly stating this is where they can find the search terms.*

 Curious about the choice to exclude studies that looked at condoms given the fact that they were included as a method choice in several of the included studies, and in fact, some of the included studies even included “traditional” methods. I don’t necessarily have an issue with it, but some justification would be appreciated.

***Response: **We have removed this phrase to avoid confusion. We excluded condoms because they do not require the same type of premeditation and planning to use them as a contraception.*

**Results**

 Data synthesis: the last sentence should be in the discussion rather than methods

***Response: **Change has been made.*

 It would be helpful to include details on the reasons/n’s for the 1425, 25, and 4 studies that were excluded after applying the inclusion/exclusion criteria at various steps. I’m a little surprised by how few studies were ultimately included and it would be useful to see why others were excluded.

***Response: **This is now included and it states: Many papers were excluded because they did not have a clear definition of intention to use (732), did not state an association between intention to use and contraceptive use (235), did not meet the study* *design* *requirements (238), did not contain sufficient information in the text to be assessed against the inclusion criteria (30), focused on condoms (161), did not include a measure of contraceptive use (61) or focused on only on the drivers of intention to use and did not test the association with actual use  (17).*

 The text states that study aim, population, location, study design, and follow-up period are included in Table 1, however, this is not the case. Please include columns for each of these variables, as well as sample sizes and titles. It would also be nice to be able to see the definition of methods used in each study. Perhaps dividing the information into 2 tables would be useful.

***Response: **We have this Table, but we see that it was not included in the paper, only Table 2 was included. Apologies and we will rectify this with the production team.*

 Suggest including full list of papers as supplemental material.

***Response:** This information is included in Table 1.*

 Without being able to see the sample sizes and follow-up periods for each paper, the statement about person-years of data is unclear. I’m assuming the statement implies just a few months of follow-up for almost 5,000 study participants. Is that correct? If not, more clarification is needed.

***Response: **We have this information in Table, but I see that it was not included in the paper, only Table 2 was included. Apologies and we will rectify this. Yes, for most data included the follow-up periods were short.*

 Associations section: the distinction between magnitude and strength of association doesn’t have a statistical meaning, so it’s unclear what is being communicated at the end of this paragraph. Why doesn’t the strength of association change after adjustment if there are examples of significant unadjusted OR and non-significant aOR *and* vice versa? Please clarify.

***Response: **Thank you for this comment but we have not changed the text because the magnitude of a coefficient, here odds ratios, indicates the direction and degree of the relationship between the predictor and outcome variable, while the strength of association indicates how precisely the coefficient is measured. What is being said here is that, as expected, when one adds more variables to the model (adjusted ORs), the magnitude of the relationship between the predictor and outcome, or independent and dependent, variable is attenuated towards zero. This is expected, as the addition of new variables typically explains additional portions of the variance in the outcome. A reduction in the statistical significance, or strength of association, indicates that adding the new variable(s) has diluted the [magnitude] of the original association between the predictor and outcome variable, so that the estimate has become less precise. So, to answer the first part of the question, what is being communicated is that there is some relationship between the added variables and intention to use, thus reducing the magnitude of the coefficients, however these coefficients continue to be very precisely estimated and are significant in the presence of effect modifiers. For the second part of the question 'Why doesn't the strength of the association change…', this is a statistical question, and the answer is that the coefficient continues to be as or close to precisely estimated in adjusted models as it is in unadjusted models.*

**Discussion**

 Appreciate the nuance about goal and implementation intentions. Is hat a distinction that was recognized by any of the excluded studies that did not assess future contraception use following report of ITU?

***Response: **Thank you for the observation and we have made the change: “None of the papers included distinguished between goal and implementation intentions.”*

 Another limitation is the variability of geographic settings for the included studies. The analysis seems to assume that the relationship between ITU and subsequent method use is generalizable across settings, which is a fairly strong assumption given the possible differences in health systems and contraceptive access. Could the wide range of ORs be explained by some of these differences?

***Response: **Thank you for the observation and we have made the change: “Geographic settings, particularly the difference in health systems and contraceptive access, may also explain the differences we found.”*

 It would be helpful to address/compare the results to unmet need. If the motivation for this study is that unmet need is an inadequate measure for predicting contraception use, how does this study compare and what do the results add? It doesn’t appear that there is currently enough evidence to support the claim that ITU is a better predictor of future use, so the conclusion seems to overstate it’s strength as a predictive measure.

***Response: **Thank you for pointing this out. We have removed the word ‘alternative’ from the conclusion as we do not draw a comparison*.
